# The impact of different distractions on outdoor visual search and object memory

**DOI:** 10.1038/s41598-023-43679-6

**Published:** 2023-10-04

**Authors:** Sarah Jasmin Nachtnebel, Alejandro Javier Cambronero-Delgadillo, Linda Helmers, Anja Ischebeck, Margit Höfler

**Affiliations:** 1 Department of Psychology, University of Graz, Universitätsplatz 2/III, 8010 Graz, Austria; 2 Department for Dementia Research, University for Continuing Education Krems, Dr.-Karl-Dorrek-Straße 30, 3500 Krems, Austria

**Keywords:** Human behaviour, Attention

## Abstract

We investigated whether and how different types of search distractions affect visual search behavior and target memory while participants searched in a real-world environment. They searched either undistracted (control condition), listened to a podcast (auditory distraction), counted down aloud at intervals of three while searching (executive working memory load), or were forced to stop the search on half of the trials (time pressure). In line with findings from laboratory settings, participants searched longer but made fewer errors when the target was absent than when it was present, regardless of distraction condition. Furthermore, compared to the auditory distraction condition, the executive working memory load led to higher error rates (but not longer search times). In a surprise memory test after the end of the search tasks, recognition was better for previously present targets than for absent targets. Again, this was regardless of the previous distraction condition, although significantly fewer targets were remembered by the participants in the executive working memory load condition than by those in the control condition. The findings suggest that executive working memory load, but likely not auditory distraction and time pressure affected visual search performance and target memory in a real-world environment.

## Introduction

Imagine you are rushing through the aisles of the supermarket, scanning the shelves for a carton of oat milk. While doing so, you might be listening to a podcast on your headphones or reciting your grocery list in your head. Maybe you suddenly realize that you are running late for an appointment, and you might have to make a quick decision to grab regular milk instead of your planned oat milk purchase. In the previous scenarios, the process of finding the oat milk is distracted in different ways, and all these distractions might lead to consequences in how you perform your search.

Visual search can be defined as the search for one or more targets within several distractors^1,2^. In laboratory settings, a common visual search task might be to search for a target letter among a set of other letters (e.g.^3–7^) or a picture amidst others (e.g.^8,9^). Previous studies have shown that different factors modulate the search process. In some laboratory searches, the target might differ from the distractors only by one relevant or salient feature (such as color) in a way that it “pops out” from its environment. Thus, it can be found immediately regardless of the number of items searched through. This is called a parallel search^10,11^. In contrast, if the target differs from the distractors by a combination of features (i.e., color and shape) and therefore, the items must be inspected one by one to find the target, the response times will increase linearly with display size. Consequently, in such serial searches, all items must be inspected if the target is absent, whereas, on average, only about half of the items need to be checked to find that the target is present. This means that search times for target-present trials are usually shorter than search times for target-absent trials (e.g.^11,12^).

The question of whether and how memory is involved during and after the search process was, and to some extent still remains a subject of debate (e.g.^5,13^). In 1998, Horowitz and Wolfe claimed memory is not involved during visual search^5^. However, in the last 20 years, it has become evident that we can use some of the gained information from the objects to improve search performance^7,14^. For instance, if participants search a display repeatedly, they are getting faster over time (e.g.^15^) or are at least capable of restricting their attention to those items relevant to the current search task (e.g.^16^). Even if participants search one display only twice, memory for previously inspected items can be observed (e.g.^3,4,6,17^). These findings have been interpreted as evidence of the involvement of short-term memory processes in visual search. Furthermore, when testing for object memory after the search has ended, it was found that searching for an object in a scene leads to a good memory representation of that object. For instance, Draschkow et al. (^18^ see also^19^) had participants search for an object or memorize an object in meaningless arrays of pictures or in real-world scenes. Their results indicated that memory for target objects was better when participants had searched for this object in a scene before than when they were instructed to explicitly memorize it. This advantage was not observed in arrays of pictures on uniform backgrounds and also not for distractors.

Previous research has already indicated some factors that might—or might not—affect visual search in terms of performance or efficiency. For instance, auditory distraction does not necessarily have a detrimental effect on visual search performance and/or accuracy. On one hand, it can be found that loud music decreased search performance in a word-search task^20^. On the other hand, listening to a podcast seems to not disrupt the detection rate for a briefly presented target (^21^ see also^22^). Similarly, white noise intensity only partly interferes with visual search, with longer response times observed at 80 dB, but shorter response times at 90 dB compared to a silent condition (^23^, see^24^, for related results).

Another type of distraction that might affect search performance is a secondary task that taxes working memory and has to be performed while the search is ongoing. For instance, Han and Kim^25^ had participants either perform an executive working memory task (i.e., counting backwards in steps of three or ordering letters alphabetically) or a control task in which information simply had to be maintained in working memory while performing a visual search task. They showed that, compared to the control task, such an executive working memory load during visual search resulted in longer response times and reduced search efficiency (for similar results, see^26,27^, Experiment 1). Also, tasks that involve visuospatial working memory load (e.g., holding the location of objects in working memory during search) have been shown to impair both search performance and search efficiency^28,29^.

Finally, a further source of search “distraction” is when the search is becoming interrupted or even aborted due to external factors. Interruptions during search seem to increase search times (e.g.^8,30,31^), although participants can quite well resume search after a brief interruption^32^. The complete abortion of a search (i.e., when the interruption occurs with no possibility to continue searching afterwards) often goes along with time pressure tasks. Time constraints have been observed to alter behavioral and oculomotor search behavior differently^33–36^. For instance, McCarley^34^ showed in a baggage-screening task that participants not only made fewer and shorter fixations when prioritizing speed over accuracy, but also missed the target more often. However, Nowakowska et al.^37^ argue that time pressure does not inherently alter visual search strategies, as they showed that irrespective of whether time constraints were present or not, most participants consistently made numerous unnecessary fixations during a visual search task. Instead, they found that a more efficient strategy emerged over time with practice.

In recent years, visual search paradigms have increasingly been transferred from artificial displays to realistic environments, such as natural scenes (e.g.^18,37–40^) or virtual reality (e.g.^19,41–43^). Traditional search paradigms and well-established results seem to successfully translate into (immersive) virtual reality (VR) environments, such that there seem to be no additional side effects that might undermine the overall outcome^43,44^. Interestingly, the results of Figueroa et al.^45^ revealed even faster response times and higher search accuracy in 3D VR than in 2D displays. Research using 3D VR showed that participants’ memory of the target location was better compared to a 2D scene^46^ with superior location memory being evident even after three days^47^. In a recent comparative study of visual search performance within analogous virtual reality (VR) and real-world environments, Van Den Oever et al.^48^ found that participants’ responses were equally as fast and accurate across the two contexts.

To date, at least to our best knowledge, there have been only a few studies that have examined visual search in the real world (e.g., searching for objects on a table^49–52^, in an indoor space^53,54^ or on grassland^55^). Among the few, Draschkow and Võ^53^ had participants either to find or to find-and-collect objects in different rooms of an apartment and showed that the amount of interaction (find vs. find-and-collect) with relevant vs. irrelevant search objects affected the identity and location memory for those objects differently. That is, while identity memory was better for the relevant than for irrelevant objects regardless of the amount of interaction, location memory was better for relevant vs. irrelevant objects only in the find-and-collect condition. This suggests that active object handling supported the prioritization of the relevant objects. However, we did not find literature on visual search in an outside environment that investigates the impact of daily distractions (such as listening to a podcast or being under time pressure) on such an outside visual search.

In the present study, we were interested to explore whether and how different types of distractions affect visual search in a natural environment. We think that our experiment can add value to the existing literature, as it expands previous research to more uncontrolled settings and bridges the gap between laboratory research and real-life situations, where distractions of all kinds are very common. We asked participants to search for objects at different university campus locations and measured both response times and search accuracy. To manipulate distraction during the search, participants either had to listen to a podcast during the search (auditory distraction), had to perform an executive working memory task, or were under time pressure during half of the searches. Furthermore, memory for present and absent targets was tested in a surprise memory test after the end of the search tasks. Based on previous findings from the laboratory, we expected that search should be faster and less accurate in target-present than in target-absent searches^11,12,56^ and present targets to be remembered better than absent targets^18,19^. Furthermore, following the findings from laboratory settings (e.g.^25^), we expected that an executive working memory load should lead to a worse search and memory performance compared to a control condition without any load. For the other two conditions (auditory distraction and time pressure), the predictions are less clear as the findings from laboratory settings are rather mixed and hardly transferable to a natural setting. Nevertheless, we assume that both listening to a podcast and time pressure should affect search accuracy and performance and lead to a worse memory for the searched-for objects.

## Method

### Participants

Sixty-one participants (all but four were students) volunteered in the experiment. Sample size using mixed-design repeated measures ANOVAs (within-between interaction) was initially determined by a conventional a priori G*Power calculation^57^. For four groups and two measurements (correlated 0.6 based on previous findings^3^) with an α of 0.05, power of 0.95 and a medium effect size of *f* = 0.3, we calculated a total sample of 44 participants (i.e., 11 participants per group). To increase the robustness of the analysis, we originally aimed for 15 participants per group. However, as it was brought to our attention by a reviewer, G*power seems to underestimate the number of participants needed given the chosen settings. When recalculating the power analysis (changing the effect size specification from the default setting “as in GPower 3.0” to “as in SPSS”; as recommended by Lakens^58^), an effect size of η^2^_p_ = 0.1 and a power of 0.80 resulted in a recommended total sample size of 108 participants. A critical discussion on this can be found in the limitation section.

Because of speed-accuracy-tradeoff (i.e., low accuracy accompanied by fast responses), the data of two participants were excluded from the analysis. 15 participants (9 female, 6 male; mean age = 23.07 years, *SD* = 3.73) were in the control group, 15 participants (11 female, 4 male; mean age = 22.6 years, *SD* = 1.96) in the auditory distraction group, 15 participants (10 female, 5 male; mean age = 22.67 years, *SD* = 2.02) in the executive working memory load group and 14 participants (10 female, 3 male, 1 non-binary; mean age = 23.14 years, *SD* = 2.41) in the time pressure group. Most of the participants (64.4%) reported that usually they are at the campus several times per week while the rest stated that they are there either once per week (20.3%), 1–2 times per month (5.1%) or that they have never been there (10.2%). Students of psychology received course credit for their participation. All participants had normal or corrected-to-normal vision, spoke fluent German and gave informed consent before the experiment. The study was approved by the ethics committee of the University of Graz and was performed in accordance with the relevant guidelines and regulations.

### Design

Participants were randomly assigned to one of four groups (control (C), auditory distraction (AD), executive working memory load (EWM) or time pressure (TP) group) and were walked to eight different locations on the main campus of the University of Graz (locations and path that was walked can be found at the Open Science Framework (OSF), see data availability section). There they had to search for ten target objects each. Half of these targets were present, while half were absent at the respective location. Participants were asked to search for the targets as fast and accurately as possible. All participants but those in the control group were either asked to perform a secondary task while searching for the target or they were asked to search under time pressure. In the auditory distraction condition, participants were required to listen to a podcast; in the executive working memory load condition, they counted backwards in steps of three, and in the time pressure condition, they were told that some of the searches would be aborted. To investigate how these different types of distractions affect visual search, response times and the accuracy of the given answers were recorded on a tablet. Furthermore, memory for the targets was tested in a surprise memory test applied after the end of the search tasks.

### Stimuli

**Figure 1:**
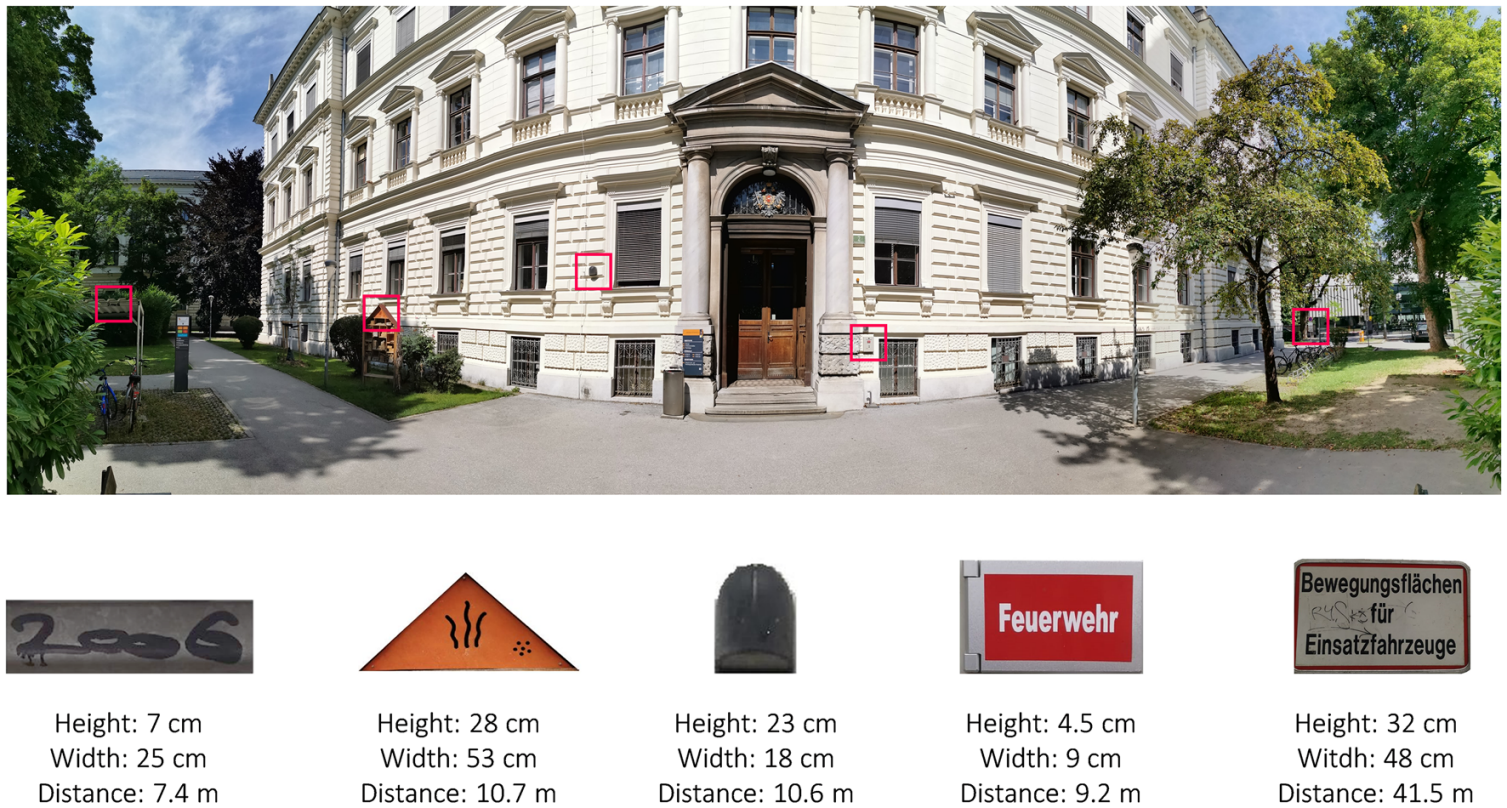
Panoramic view of the first location “Psychology Building” with the respective present targets, as well as their position marked, height, width, and distance to the participant.

For the target-present trials, 40 photographs of objects (five at each location) were taken at eight different locations on the Campus of the University of Graz (e.g., in front of the main entrance of the Main University Building or in front of the University’s Main Library). Pictures included objects such as sculptures, road signs, stickers, or graffiti that were all exclusive to each location. All objects were kept at their “natural location”, which means that we did not add objects but took photographs of objects that already existed on campus. All locations included targets that were in the immediate surroundings of the participants, but also objects that were further away. An example of the five present targets for the first location, including their size and distance from the participant can be found in Fig. 1. All other locations, as well as their absent and present targets can be found at the OSF (see data availability section). Furthermore, 60 pictures were taken at other places outside of the campus. 40 of them were randomly assigned to the eight locations of the campus as absent targets (five at each location), and 20 acted as foils for the memory test conducted after the end of the experiment. We removed the background of the pictures with *GIMP*^59^. Stimuli were shown in color on a white background on a handheld Android tablet. The experiment was programmed on *PsychoPy*^60^ and uploaded to *Pavlovia*^61^ for online access on the tablet. The language of instruction was German. For the auditory distraction condition, a true crime podcast was chosen for participants to listen to. The 43 min and 28 s long episode was divided into 8 snippets for the eight locations.

### Procedure

**Figure 2:**
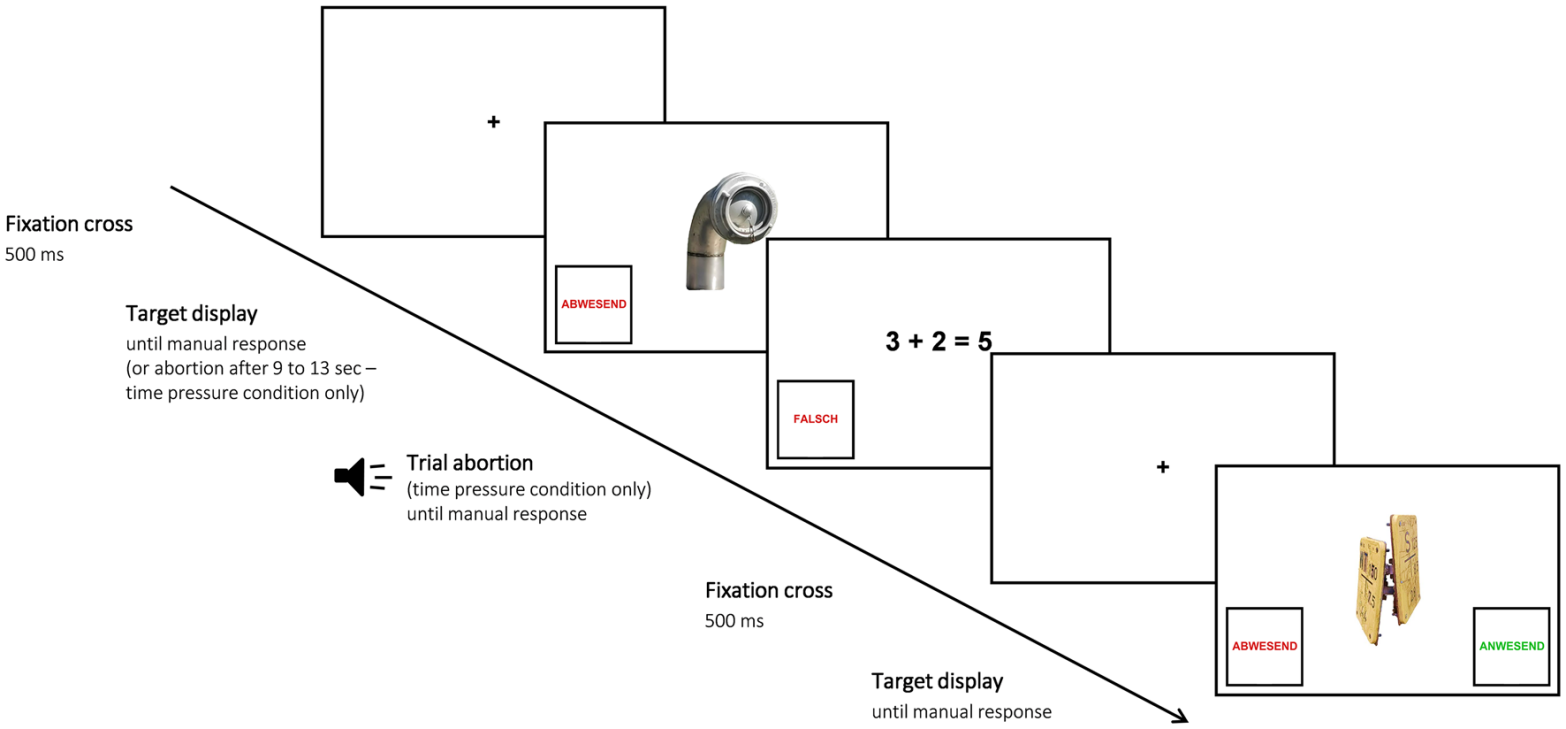
Procedure of two trials in the time pressure condition at the location “University Library” as presented on the tablet screen. The first target is absent, which is why (only in the time pressure condition) a trial abortion would occur after 9 to 13 s. The second target is present, and therefore the target display is presented until manual response.

The eight different search locations on the University campus were connected via an approximately one-kilometer-long walk. Every participant was walked to each location and placed on the same spot which assured that all targets were visible from their point-of-view. They then received a tablet on which the search task would be presented. Participants were instructed to search for 10 different targets in a 180° radius. They were free to move their head but were asked not to turn their whole body around. Every trial started with the presentation of a fixation cross in the center of the tablet screen for 500 ms, followed by the search target (see Fig. 2). Participants had to search for the target object and to give a manual absent or present response by tapping a button on the left or right bottom of the display with their finger. Following this, a new trial started. No additional tasks were included if the participant was in the control group and no additional tasks were performed during the walks between the locations, regardless of the condition. All searches at one location took around 2–3 min on average.

In the auditory distraction condition, participants were asked to listen to a podcast while searching for the targets. All participants were asked to bring headphones which were plugged into a mobile phone playing the podcast. Before starting the search, the podcast was started with a button press by the experimenter. If, on rare occasions (30 out of 1200 trials), participants took longer than the respective podcast sequence, they were instructed to keep searching until finished.

In the executive working memory load condition, similarly to Han and Kim^25^ a unique three-digit number was presented on the display before the start of the first search at the respective campus location. Participants were asked to count down aloud in steps of three from the shown number while searching for the objects. The number was presented in the display center for five seconds, and participants were allowed to start counting down as soon as they saw it. The experimenter noted down every number that was said.

In the time pressure condition, participants were informed that sometimes they would not be able to finish their search. Trial abortions were programmed to occur after 9—13 s in all but four target-absent trials (36 trials). In four target-absent trials (at four different campus locations), participants were allowed to finish their search to avoid learning effects. Each search abortion started with a tone (1,000 ms) coming from the tablet, followed by the presentation of an easy mathematical equation in the format A + B = C or A − B = C, which had to be classified as wrong or right by the participant by tapping a button for incorrect (“Falsch”) or correct (“Richtig”) on the bottom left and right side of the display, respectively (see Fig. 2). This task was chosen to ensure that the participants reliably stopped searching and looked back at the screen. To prevent that participants accidentally gave a delayed response meant for the search task although the trial abortion display was already on, the buttons only appeared after the tone. 18 equations were correct, and 18 equations were incorrect. All numbers were single digits. No equation was shown twice during the experiment. After the button press, a new trial started with the presentation of the fixation cross and a new target.

After the eighth campus location, participants were asked to complete a memory test. In this test, we subsequently presented all the objects they had to search for from the task before on the tablet, as well as 20 more pictures they did not have to search for (foils). Participants were asked to tap a button for yes (“Ja”), no (“Nein”) and I do not know (“Ich weiß nicht”), on the right side of the display depending on whether they had previously searched for the object or not (question 1). If the participants answered “no” or “I do not know”, the next object was presented. If the answer was “yes” they were asked at which location on the campus they had to search for the object (question 2). To this end, pictures of all eight locations, including their names and an additional “I do not know” button were presented on the tablet and participants were asked to tap on the correct picture.

Finally, when the memory test on the tablet was completed, all participants of the time pressure condition were asked how disruptive they found the abortion of the search on a scale from one to seven, with one being not disruptive at all and seven being extremely disruptive. The participants of the auditory distraction condition were required to answer nine short retention questions regarding the podcast they had heard before. Questions were chosen to be about topics that were mentioned at the very beginning of the respective sequence to ensure all participants heard them. Finally, all participants were handed a blank sheet of paper and asked to draw a map of the campus with all the buildings and mark the locations of the search and the path. The results of this task are not included in the current paper. Overall, the experiment lasted about an hour on average.

## Results

In total, we collected 4720 trials. 37 trials were removed because of unexpected disruptions during the search task (i.e., incoming traffic or passersby). 30 trials where the participants took longer than the respective podcast sequence were excluded. Further 247 trials had to be excluded from analysis because the respective target objects were removed from the location or were hidden by another object at the time of testing. In total this amounts to 314 trials (6.7%). Additionally, 75 trials could not be analyzed for the search task because of the loss of the data of one participant due to technical issues. All analysis were performed with *Jamovi*^62^. The alpha level was set to 0.05.

For the auditory distraction condition, 10 out of 15 podcast memory tests were available for analysis. On average 66.4% (*SD* = 26.6%) of the questions were solved correctly. For the time pressure condition 333 abortions occurred. Participants solved the equation correctly in 89.9% (*SD* = 3.7%) of the cases on average. 10 out of 14 participants answered the question on how disruptive they found the abortion of the search on a scale from one to seven, with one being not disruptive at all and seven being extremely disruptive. On average participants found the abortions to be moderately disruptive (*M* = 3.8, *SD* = 1.81).

### Visual search

For the analysis of the search performance in the time pressure condition, we were only able to analyze the catch-trials of the time pressure condition and the trials where participants gave an answer before the abortion occurred. This amounts to 40.5% of the absent searches of the time pressure condition, which is 227 trials.

#### Error rates

**Figure 3:**
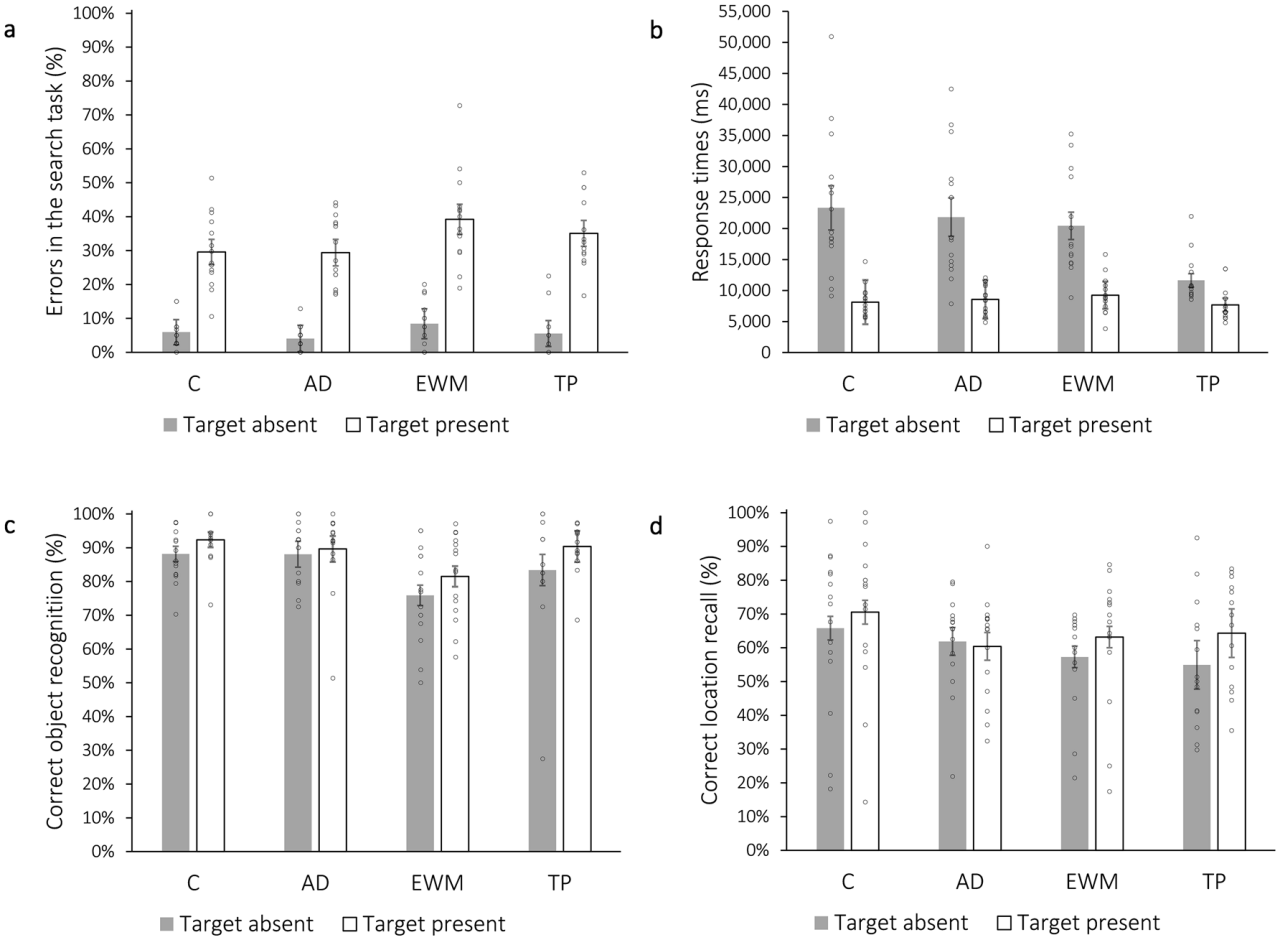
(**a**) Errors in the search task (%), (**b**) Response times (ms), (**c**) Object recognition (%), (**d**) Location recall (%) divided by distraction condition (*C* control condition, *AD* auditory distraction condition, *EWM* executive working memory load condition, *TP* time pressure condition), as well as target presence. Error bars represent 95% confidence intervals^63,64^. Circles mark individual participants.

Overall, error rates seemed to be higher for target-present than for target-absent searches, and higher for the executive working memory load condition than the other distraction conditions (see Fig. 3a). To test if the error rates differ significantly between groups, we performed a 2 × 4 mixed ANOVA with target presence (absent vs. present) as a within-subject factor and distraction condition (control, auditory distraction, executive working memory load, time pressure) as a between-subject factor. The error rates in the target-present searches were significantly higher than the error rates in the target-absent searches, *F*(1, 54) = 365.18; *p* < 0.001; η^2^_p_ = 0.871. There was also a main effect of distraction condition, *F*(3, 54) = 3.02; *p* = 0.038; η^2^_p_ = 0.144. The interaction was not significant, *F*(3, 54) = 1.41; *p* = 0.249; η^2^_p_ = 0.073. Bonferroni-corrected post-tests revealed that the error rates of the executive working memory load condition were significantly higher than the error rates of the auditory distraction condition (*p* = 0.046). No other differences were significant, AD—C: *p* = 1.00; AD—TP: *p* = 1.00; C—EWM: *p* = 0.132; C—TP: *p* = 1.00; EWM—TP: = 1.00.

On average present targets were missed in 33.95% (*SD* = 24.79%) of the cases. Absent targets were falsely recognized as present in 5.97% (*SD* = 10.16%) of the cases. 9 of the 38 targets included in the analysis were not found by at least half of the participants. 13 of those 38 targets were found by at least 75% of the participants. Targets that were particularly hard to find were for example a flag indicating roof avalanches on a building down the street (89.66% errors) or the Letter “E” in the labeling on the façade of the chemics institute (91.07% errors). Individual targets that were found by all participants were a big statue and a bright yellow mailbox. In depth by-item performance can be found at the OSF (see data availability section). In order to preserve the ecological validity of our study, no targets were excluded from the analysis due to high error rates.

#### Search times

Figure 3b provides the response times separately for target absent and present searches and for the distraction conditions. Descriptively, search times were higher for absent than for present searches in all but the time pressure condition. Since, in that condition, target-absent searches were aborted after 9–13 s, responses in the absent trials could only be made before this point. Hence, the average response times for target-absent searches were almost as fast as for target-present searches. Because of this reason, all trials from the time pressure condition were excluded from the following analysis.

To test if the response times differ significantly between the three distraction conditions (with the time pressure condition not being included in this analysis), we performed a 2 × 3 ANOVA: The response time (control, auditory distraction, executive working memory load) of the absent searches was longer than of the present searches, *F*(1, 41) = 109.07; *p* < 0.001; η^2^_p_ = 0.727. There was no main effect of distraction condition, *F*(2, 41) < 1; *p* = 0.922; η^2^_p_ = 0.004, and there was no interaction, *F*(2, 41) < 1; *p* = 0.444; η^2^_p_ = 0.039.

Furthermore, there was also an overall effect of trial number on the search time (averaged across target presence): Spearman correlations for each condition separately showed on average a significant decrease in search time as the trial number increases, *r*_C_(8) = − 0.78, *p* = 0.012; *r*_AD_(8) = − 0.71, *p* = 0.028; *r*_EWM_(8) = − 0.70, *p* = 0.031; *r*_TP_(8) = − 0.67, *p* = 0.039. This suggests that participants retained information from previous trials to improve further searches.

### Memory test

All means and individual data points are shown in Fig. 3c,d. Foils were on average only falsely recognized as targets in 5% (*SD* = 6.55%) of the cases in the control condition, in 2.67% (*SD* = 2.58%) in the auditory distraction condition, in 6% (*SD* = 9.1%) in the executive working memory load condition and in 2.86% (*SD* = 3.78%) of the cases in the time pressure condition. A one-way ANOVA shows that the difference between the groups is not significant, *F*(3, 55) = 1.07, *p* = 0.371.

To examine the effect of distractions on object memory and location memory, we conducted two 2 × 4 ANOVAs, respectively, with target presence as a within-subjects factor (absent vs. present) and the distraction condition as a between-subjects factor. Present targets were significantly better recognized than absent targets, *F*(1, 55) = 13.15, *p* < 0.001, η^2^_p_ = 0.193. There was also a main effect of distraction condition *F*(3, 55) = 3.67; *p* = 0.018; η^2^_p_ = 0.167. The interaction was not significant, *F*(3, 55) < 1; *p* = 0.482; η^2^_p_ = 0.043. Bonferroni-corrected post-tests revealed that the recognition of the targets in the executive working memory load condition was significantly lower than in the control condition (*p* = 0.023). No other differences were significant, AD—C: *p* = 1.00; AD—EWM: *p* = 0.061; AD—TP: *p* = 1.00; C—TP: *p* = 1.00; EWM—TP: *p* = 0.238.

Similarly to the object memory, the locations (question 2) of the targets that were present were remembered significantly more often than the location of targets that were absent, *F*(1, 55) = 7.57, *p* = 0.008, η^2^_p_ = 0.121). However, the condition seems to not modulate location memory for targets: there was no main effect of distraction condition, *F*(3, 55) < 1; *p* = 0.515; η^2^_p_ = 0.040, and no interaction, *F*(3, 55) = 1.79; *p* = 0.160; η^2^_p_ = 0.089.

When excluding those trials of the search task that were solved incorrectly from the analysis of the memory tasks, the main results could be replicated. Means and standard deviations can be found at the OSF (see data availability section). Present targets were still significantly better recognized than absent targets, *F*(1, 55) = 21.61, *p* < 0.001, η^2^_p_ = 0.282. There was a main effect of distraction condition *F*(3, 55) = 3.08; *p* = 0.035; η^2^_p_ = 0.144. The interaction was not significant, *F*(3, 55) = 1.02; *p* = 0.392; η^2^_p_ = 0.053. Bonferroni-corrected post-tests revealed none of the groups differed significantly, although tendencies stayed the same, AD—C: *p* = 1.00; AD—EWM: *p* = 0.079; AD—TP: *p* = 1.00; C—EWM: *p* = 0.059; C—TP: *p* = 1.00; EWM—TP: *p* = 0.350. The locations (question 2) of the targets that were present were remembered significantly more often than the location of targets that were absent, *F*(1, 55) = 25.80, *p* < 0.001, η^2^_p_ = 0.319), there was no main effect of distraction condition, *F*(3, 55) < 1; *p* = 0.641; η^2^_p_ = 0.030, and no interaction, *F*(3, 55) = 1.85; *p* = 0.149; η^2^_p_ = 0.092.

## Discussion

We investigated the effects of different kinds of distractions during a visual search in a real-world environment on search performance and memory for the searched objects. Participants searched for 80 targets (half of them present) at eight different locations on a university campus. They were either tasked to search without any distraction (control condition), while listening to a podcast (auditory distraction condition), while counting down aloud in intervals of three (executive working memory load condition), or while searching under time pressure (time pressure condition). Regardless of the distraction condition, search time was longer, and fewer errors were made in target-absent than in target-present trials. Furthermore, search performance was seemingly impaired particularly in the executive working memory load condition. Error rates in the search task were higher in this condition than in the auditory distraction condition. The executive working memory load also led to a worse memory performance for targets compared to the control condition. Overall, object- and location memory were better for targets that had been present during the search tasks, compared to memory for absent targets.

The finding that search time was longer and fewer errors were made in target-absent than target-present searches reflects a basic finding within standard visual-search tasks conducted in laboratory settings or virtual-world environments (e.g.^11,12,65,66^). Interestingly, the error rates were overall high in target-present searches (ranging from 29 to 39% on average) and therefore higher than one would expect from laboratory settings. These high error rates cannot be attributed to an overall lack of search accuracy, since the target-absent searches were completed very accurately, with an average of 4–8% errors. Therefore, we did not exclude participants with a high amount of target misses because they did not report false alarms at an above chance level and did not show a speed-accuracy tradeoff. This approach is also justified as there is anecdotal evidence from the direct observations of the experimenters accompanying the participants that almost all of the participants took the tasks very seriously. The high rate of target misses likely stem from the target objects being differently difficult to find, such that some (usually far away) targets were found rarely (around 17% of the targets were not found by at least half of the participants). Still, we chose to keep all targets in our analysis, due to the fact that our research was conducted in a real-world context, which is inherently dynamic and characterized by a great degree of variability and complexity. Together, these observations suggest that the elevated error rates are linked to the challenges of locating targets in a complex naturalistic setting with multiple additional irritants, such as people walking by, traffic noises or odd smells while in some instances also being engaged in a secondary task. Similarly, when letting participants search for coins on a grassland Riggs et al.^55^ also found error rates up to 55%, indicating that target misses could most likely be a result of this complexity of surroundings in the real world.

We also found that absent targets were generally remembered worse than present targets, regardless of the distraction condition. This is in line with previous findings that showed that present targets are remembered better than all the other objects in the scene^18,19^. It is also important to note that the semantics of the scene might have played a crucial role here. Participants saw a present target embedded in the correct semantic scene, and hence, memory representation for the respective location might have also been increased in contrast to a situation in which they would have just viewed the picture on the tablet without any kind of natural background (^18,19,40,67–70^ see however^71^). However, we used a recognition test in order to assess the object memory. With this test we cannot rule out that participants only remembered the images instead of the actual objects as the objects were presented as picture cues during the search task. Hence, for future research it might be advisable to use verbal cues instead of picture cues for the search tasks.

Although the error rates were highest in the executive working memory load condition and memory for the target objects was less pronounced in this condition, search time was not increased significantly compared to the other conditions. This result contrasts previous findings that showed that executive working memory load led to a decrease in search performance (e.g.^25,26^). However, when Li et al.^27^ investigated the effect of no, low, and high executive working memory loads on visual search in a more similar way, using natural scenes, they also found no effect of executive working memory load on search performance (but also not on accuracy) when the target was presented as a picture and not as a word stimulus before the trial. They argued that the picture might have provided better guidance than a word cue in the load conditions, which might also be valid for the current experiment (see also, e.g.^38^). Nevertheless, it remains open whether and how such possible effect of guidance of the picture template might have affected the other search conditions.

Neither auditory distraction nor time pressure did significantly affect search accuracy or performance or led to a decreased object or location memory in the memory test in our experiment. This finding for auditory distractions contradicts some previous findings that showed an effect of for example, loud music played via headphones or listening to white noise on search performance^20,23^. However, in those studies both the search tasks (i.e., searching for letters or words) and the auditory distractions might have relied on verbal working memory resources, which might have resulted in interference effects on the visual search tasks. In our case, we provided pictures as search templates and therefore, the capacity of verbal working memory might have not been exceeded by the requirements of the search tasks. Accordingly, in a more similar study to ours, Spence et al.^21^ showed, in a laboratory setting using non-verbal stimuli that neither active nor passive listening to a podcast increased error rates or search times compared to a “silence” condition. Similar results were observed by Shinohara et al.^22^ who found that listening to audio clips, such as traffic information, radio news, a talk show, or a weather forecast did not elongate response times compared to a control condition. Also in this case, the search task (searching for a complete circle among incomplete ones) might have not interfered with contents from verbal working memory. Furthermore, it seems to be reasonable that attention has to be regularly shifted from one task to the other in a real-world environment (e.g., if you have troubles finding the oat milk in the grocery store while listening to a podcast you might use more resources for the search task and then switch back to listening again). This was easily possible in the current experiment, because, compared to the executive working memory task, in which participants had to speak aloud, in the auditory distraction condition participants might have lost concentration on the podcast. This is also evident, when taking into consideration that on average, only about 66% of the rather easy podcast memory test questions were solved correctly.

The finding for the time pressure condition differs from conclusions drawn in earlier studies that showed that searching while being time-constrained leads to less accurate responses^33^. Although, in our experiments, error rates were overall high in target-present searches, adding time pressure did not lead to a further increase of the error rates. One explanation for our results could be that the participants were able to adapt their behavior in response to the time constraint, allowing them to successfully locate the target. This possibility is further supported by the findings of McCarley^34^, who showed that, when participants emphasized speed of search, they made fewer and shorter fixations (thereby increasing their search speed), but were still able to recognize a target upon fixation.

There are some limitations in the current study. First and foremost, we want to address that the insignificant results between the different groups (control, auditory distraction, executive working memory load, time pressure) may be due to a reduced power and therefore could be a result of type II errors (i.e., false negatives). The default calculation of G*power does not appear to account for the correlation among measures which leads to errors for power analyses that include within subject factors^72^. A sensitivity analysis in G*power revealed that our two-way ANOVA with 59 participants over four groups and two measurements would be sensitive to effects of *f(U)* = 0.46 with 80% power (α = 0.05). This implies that the study would not be able to reliably detect effects smaller than η^2^_p_ = 0.17. However, collecting data of more participants was not feasible, because the original environment had changed due to construction work. Such a reduced control of the experimental setup is detrimental to reproducibility and should be considered when designing experiments in real-world environments. Hence, in light of the reduced power of the current findings, the results should be interpreted rather cautiously. In future studies, using a full within-subject design instead of a mixed design would enhance statistical power. The visual search environment was also less stable across the study than a visual search in the laboratory would have been. That is, varying weather conditions and even seasonal effects might have affected the search tasks such that some of the stimuli might have been easier to detect under specific environmental conditions^73^. Also, some of the targets were removed by third parties during the study or were occasionally hidden by other objects during the search task. A second factor we could not control for was the actual search behavior, as indicated by the eye movements of the participants, which could be observed using mobile eye-tracking devices in future studies. Especially the question of whether and how participants shift their search criterion and produce much more target misses than expected might be answered by this technique. Additionally, we observed a decrease in search time as the number of trials increased. We believe this is likely due to participants forming a mental representation of the visual scene that could have enhanced their ability to optimize their search strategy over time^3^. Eye-tracking could shed light on this process by visualizing the participants' focus shifts and attention distribution, thereby revealing how their mental representation evolves and aids in search optimization. Additionally, most of our participants were on campus at least once per week (85%), which could have influenced the outcome of our experiment, even though we carefully selected most targets to be inconspicuous, even if walked past every day. Students of different fields of study might have different familiarity levels with the specific locations we used in our experiment. Controlling for this variable would have been possible, if we had asked about how familiar they were with the locations we visited. Lastly, we did not allow movement beyond the 180° head-rotation. This was necessary to make sure every participant was in a position where they could see all targets, but constrains our results again to a controlled setting.

While searching in the real world differs greatly from searching in the tightly controlled conditions from experiments carried out in the laboratory or complex virtual environments^74^, our results confirm previous findings from these settings in terms of visual search and memory performance for the searched objects and extended them to the outside world. We gained valuable insights into how different types of distractions might impact visual search and memory performance.

## Data Availability

Data, analysis scripts and materials of this study are available as supplementary material at the Open Science Framework (10.17605/OSF.IO/54SU3).

## References

[1] Eckstein MP (2011). Visual search: A retrospective. J. Vis..

[2] Wolfe JM (2020). Visual search: How do we find what we are looking for?. Annu. Rev. Vis. Sci..

[3] Höfler M, Gilchrist ID, Körner C (2014). Searching the same display twice: Properties of short-term memory in repeated search. Atten. Percept. Psychophys..

[4] Höfler M, Gilchrist I, Körner C (2015). Guidance toward and away from distractors in repeated visual search. J. Vis..

[5] Horowitz TS, Wolfe JM (1998). Visual search has no memory. Nature.

[6] Körner C, Gilchrist ID (2007). Finding a new target in an old display: Evidence for a memory recency effect in visual search. Psychon. Bull. Rev.

[7] Kristjánsson Á (2000). In search of remembrance: Evidence for memory in visual search. Psychol. Sci..

[8] Alonso D, Lavelle M, Drew T (2021). The performance costs of interruption during visual search are determined by the type of search task. Cogn. Res..

[9] Yang H, Zelinsky GJ (2009). Visual search is guided to categorically-defined targets. Vis. Res..

[10] Treisman A (1988). Features and objects: The fourteenth bartlett memorial lecture. Q. J. Exp. Psychol. Sect. A.

[11] Treisman AM, Gelade G (1980). A feature-integration theory of attention. Cogn. Psychol..

[12] Scialfa CT, Joffe KM (1998). Response times and eye movements in feature and conjunction search as a function of target eccentricity. Percept. Psychophys..

[13] Wolfe JM, Horowitz TS (2017). Five factors that guide attention in visual search. Nat. Hum. Behav..

[14] Beck MR, Peterson MS, Boot WR, Vomela M, Kramer AF (2006). Explicit memory for rejected distractors during visual search. Vis. Cogn..

[15] Hout MC, Goldinger SD (2012). Incidental learning speeds visual search by lowering response thresholds, not by improving efficiency: Evidence from eye movements. J. Exp. Psychol. Hum. Percept. Perform..

[16] Kunar MA, Flusberg S, Wolfe JM (2008). The role of memory and restricted context in repeated visual search. Percept. Psychophys..

[17] Körner C, Höfler M, Ischebeck A, Gilchrist I (2018). The consequence of a limited-capacity short-term memory on repeated visual search. Vis. Cogn..

[18] Draschkow D, Wolfe JM, Vo ML-H (2014). Seek and you shall remember: Scene semantics interact with visual search to build better memories. J. Vis..

[19] Helbing J, Draschkow D, Võ ML-H (2020). Search superiority: Goal-directed attentional allocation creates more reliable incidental identity and location memory than explicit encoding in naturalistic virtual environments. Cognition.

[20] Radhakrishnan A, Balakrishnan M, Behera S, Raghunandhan R (2022). Role of reading medium and audio distractors on visual search. J. Optom..

[21] Spence I, Jia A, Feng J, Elserafi J, Zhao Y (2013). How speech modifies visual attention: Speech and visual attention. Appl. Cognit. Psychol..

[22] Shinohara K, Nakamura T, Tatsuta S, Iba Y (2010). Detailed analysis of distraction induced by in-vehicle verbal interactions on visual search performance. IATSS Res..

[23] Warner HD, Heimstra NW (1972). Effects of noise intensity on visual target-detection performance. Hum. Factors.

[24] Taylor W, Melloy B, Dharwada P, Gramopadhye A, Toler J (2004). The effects of static multiple sources of noise on the visual search component of human inspection. Int. J. Ind. Ergon..

[25] Han S-H, Kim M-S (2004). Visual search does not remain efficient when executive working memory is working. Psychol. Sci..

[26] He J, McCarley JS (2010). Executive working memory load does not compromise perceptual processing during visual search: Evidence from additive factors analysis. Atten. Percept. Psychophys..

[27] Li W, Guan J, Shi W (2021). Increasing the load on executive working memory reduces the search performance in the natural scenes: Evidence from eye movements. Curr. Psychol..

[28] Oh SH, Kim MS (2004). The role of spatial working memory in visual search efficiency. Psychon. Bull. Rev..

[29] Woodman GF, Luck SJ (2004). Visual search is slowed when visuospatial working memory is occupied. Psychon. Bull. Rev.

[30] Brazzolotto P, Michael GA (2021). Do not interrupt me if it makes me feel something: Study of the effect of the pleasantness of interruptions on performance. Eur. Rev. Appl. Psychol..

[31] Williams LH, Drew T (2017). Distraction in diagnostic radiology: How is search through volumetric medical images affected by interruptions?. Cogn. Res..

[32] Lleras A, Rensink RA, Enns JT (2005). Rapid resumption of interrupted visual search: New insights on the interaction between vision and memory. Psychol. Sci..

[33] Rieger T, Heilmann L, Manzey D (2021). Visual search behavior and performance in luggage screening: Effects of time pressure, automation aid, and target expectancy. Cogn. Res. Princ. Implic..

[34] McCarley JS (2009). Effects of speed–accuracy instructions on oculomotor scanning and target recognition in a simulated baggage X-ray screening task. Ergonomics.

[35] Rieger T, Manzey D (2022). Understanding the impact of time pressure and automation support in a visual search task. Hum. Factors.

[36] Yu R, Yang L, Guo X, Zhang Y (2015). Effect of time pressure on dynamic visual search performance. Procedia Manuf..

[37] Nowakowska A, Clarke ADF, Von Seth J, Hunt AR (2021). Search strategies improve with practice, but not with time pressure or financial incentives. J. Exp. Psychol. Hum. Percept. Perform..

[38] Malcolm GL, Henderson JM (2009). The effects of target template specificity on visual search in real-world scenes: Evidence from eye movements. J. Vis..

[39] Nuthmann A (2014). How do the regions of the visual field contribute to object search in real-world scenes? Evidence from eye movements. J. Exp. Psychol. Hum. Percept. Perform..

[40] Võ ML-H, Wolfe JM (2013). Differential electrophysiological signatures of semantic and syntactic scene processing. Psychol. Sci..

[41] Beitner J, Helbing J, Draschkow D, Võ ML-H (2021). Get your guidance going: Investigating the activation of spatial priors for efficient search in virtual reality. Brain Sci..

[42] David EJ, Beitner J, Võ ML-H (2021). The importance of peripheral vision when searching 3D real-world scenes: A gaze-contingent study in virtual reality. J. Vis..

[43] Olk B, Dinu A, Zielinski DJ, Kopper R (2018). Measuring visual search and distraction in immersive virtual reality. R. Soc. Open Sci..

[44] Foerster RM, Poth CH, Behler C, Botsch M, Schneider WX (2016). Using the virtual reality device Oculus Rift for neuropsychological assessment of visual processing capabilities. Sci. Rep..

[45] Figueroa JCM, Arellano RAB, Calinisan JME, Cassenti DN (2018). A comparative study of virtual reality and 2D display methods in visual search in real scenes. Advances in Human Factors in Simulation and Modeling.

[46] Li C-L, Aivar MP, Kit DM, Tong MH, Hayhoe MM (2016). Memory and visual search in naturalistic 2D and 3D environments. J. Vis..

[47] Kit D, Katz L, Sullivan B, Snyder K, Ballard D, Hayhoe M (2014). Eye movements, visual search and scene memory, in an immersive virtual environment. PLoS ONE.

[48] Van Den Oever, F., Gorobets, V., Saetrevik, B., Fjeld, M. & Kunz, A. Comparing Visual Search between Physical Environments and VR. In *2022 IEEE International Symposium on Mixed and Augmented Reality Adjunct (ISMAR-Adjunct)* 411–416 (IEEE, 2022).

[49] Howard CJ, Pharaon RG, Körner C, Smith AD, Gilchrist ID (2011). Visual search in the real world: Evidence for the formation of distractor representations. Perception.

[50] Mack SC, Eckstein MP (2011). Object co-occurrence serves as a contextual cue to guide and facilitate visual search in a natural viewing environment. J. Vis..

[51] Ramzaoui H, Faure S, Spotorno S (2021). Age-related differences when searching in a real environment: The use of semantic contextual guidance and incidental object encoding. Q. J. Exp. Psychol..

[52] Sauter M, Stefani M, Mack W (2020). Towards interactive search: Investigating visual search in a novel real-world paradigm. Brain Sci..

[53] Draschkow D, Võ MLH (2016). Of, “what” and “where” in a natural search task: Active object handling supports object location memory beyond the object’s identity. Atten. Percept. Psychophys..

[54] Foulsham T, Chapman C, Nasiopoulos E, Kingstone A (2014). Top-down and bottom-up aspects of active search in a real-world environment. Can. J. Exp. Psychol..

[55] Riggs CA, Cornes K, Godwin HJ (2017). The importance of search strategy for finding targets in open terrain. Cogn. Res..

[56] Wolfe JM (1998). What can 1 million trials tell us about visual search?. Psychol. Sci..

[57] Faul F, Erdfelder E, Lang A-G, Buchner A (2007). G*Power 3: A flexible statistical power analysis program for the social, behavioral, and biomedical sciences. Behav. Res. Methods.

[58] Lakens D (2013). Calculating and reporting effect sizes to facilitate cumulative science: A practical primer for t-tests and ANOVAs. Front. Psychol..

[59] The GIMP Development Team. *GIMP.* (2019).

[60] Peirce J (2019). PsychoPy2: Experiments in behavior made easy. Behav. Res..

[61] Bridges D, Pitiot A, MacAskill MR, Peirce JW (2020). The timing mega-study: Comparing a range of experiment generators, both lab-based and online. PeerJ.

[62] The Jamovi Project. *jamovi*. (Version 2.3) [Computer Software]. Retrieved from https://www.jamovi.org (2022).

[63] Cousineau D (2005). Confidence intervals in within-subject designs: A simpler solution to Loftus and Masson’s method. Tutor. Quant. Methods. Psychol..

[64] Morey RD (2008). Confidence intervals from normalized data: A correction to Cousineau (2005). Tutor. Quant. Methods. Psychol..

[65] Ward, A. R. & Capra, R. Immersive search: Using virtual reality to examine how a third dimension impacts the searching process. In *Proceedings of the 43rd International ACM SIGIR Conference on Research and Development in Information Retrieval* 1621–1624 (ACM, 2020).

[66] Zenger B, Fahle M (1997). Missed targets are more frequent than false alarms: A model for error rates in visual search. J. Exp. Psychol. Hum. Percept. Perform..

[67] Hayes SM, Nadel L, Ryan L (2007). The effect of scene context on episodic object recognition: Parahippocampal cortex mediates memory encoding and retrieval success. Hippocampus.

[68] Hollingworth A, Henderson JM (2002). Accurate visual memory for previously attended objects in natural scenes. J. Exp. Psychol. Hum. Percept. Perform..

[69] Hollingworth A (2006). Scene and position specificity in visual memory for objects. J. Exp. Psychol. Learn. Mem. Cogn..

[70] Josephs EL, Draschkow D, Wolfe JM, Võ ML-H (2016). Gist in time: Scene semantics and structure enhance recall of searched objects. Acta Psychol..

[71] Evans KK, Wolfe JM (2022). Sometimes it helps to be taken out of context: Memory for objects in scenes. Vis. Cogn..

[72] Lakens D, Caldwell AR (2021). Simulation-based power analysis for factorial analysis of variance designs. Adv. Methods Pract. Psychol. Sci..

[73] Adams WJ (2007). A common light-prior for visual search, shape, and reflectance judgments. J. Vis..

[74] Clark K, Cain MS, Adamo SH, Mitroff SR, Dodd MD, Flowers JH (2012). Overcoming hurdles in translating visual search research between the lab and the field. The Influence of Attention, Learning, and Motivation on Visual Search.

